# *FeatureViewer*, a BioJS component for visualization of position-based annotations in protein sequences

**DOI:** 10.12688/f1000research.3-47.v2

**Published:** 2014-04-09

**Authors:** Leyla Garcia, Guy Yachdav, Maria-Jesus Martin

**Affiliations:** 1 European Bioinformatics Institute EMBL-EBI, Hinxton, Cambridge, CB10 1SD, UK; 2 TUM, Department of Informatics, Bioinformatics and Computational Biology-I12, 85748 Garching, Germany

## Abstract

**Summary: **FeatureViewer is a BioJS component that lays out, maps, orients, and renders position-based annotations for protein sequences. This component is highly flexible and customizable, allowing the presentation of annotations by rows, all centered, or distributed in non-overlapping tracks. It uses either lines or shapes for sites and rectangles for regions. The result is a powerful visualization tool that can be easily integrated into web applications as well as documents as it provides an export-to-image functionality.

**Availability:** https://github.com/biojs/biojs/blob/master/src/main/javascript/Biojs.FeatureViewer.js; http://dx.doi.org/10.5281/zenodo.7719

## Introduction

Position-based annotation is one of the cornerstones of bioinformatics. A great number of databases, analysis and prediction methods are geared towards providing data mapped to specific sequence coordinates. In the case of proteins, the Pfam ^1^ database identifies, marks-up, and characterizes different functional regions within a given protein. The coordinates of these domains are often given in terms of the start and end position within the protein. The largest pool of reviewed and automatically annotated proteins is provided by the UniProt Consortium ^2^. It contains position-based annotations for structural regions, modified residues, and functional sites among others. Finally, protein feature prediction methods such as those integrated into PredictProtein ^3^ provide position-based annotations such as secondary structure states, buried and exposed residues, coiled-coil stretches, and disordered regions. PredictProtein also maps functional regions such as protein-protein binding sites and protein-DNA binding sites onto positions within the sequence.

**Figure 1.**
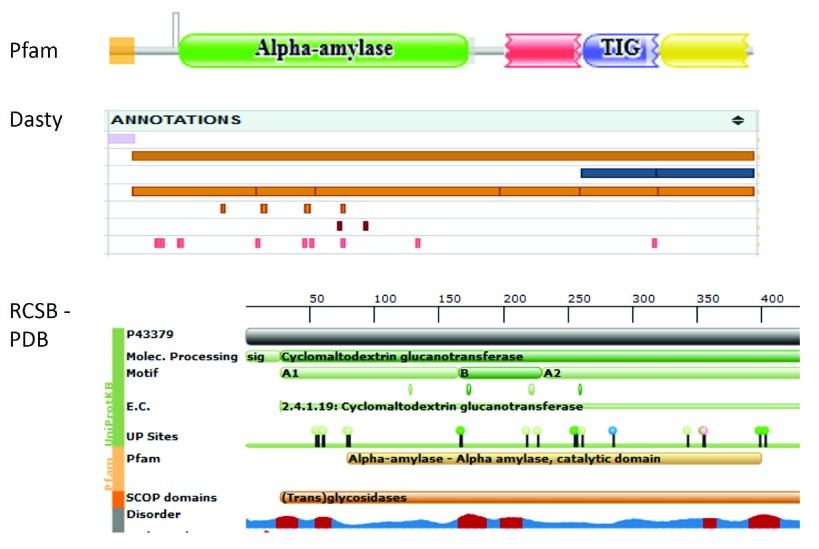
Visualization of a portion of the protein P43379–UniProt accession, in Pfam, Dasty and RCSB-PDB.

Visualization of protein sequence features has already been used in different projects, some of which are shown in Figure 1. For intance, Pfam renders Pfam domains as well as some sites of interest, such as metals, active binding sites, and also disulphide bonds. It supports uncertainty for the start and end positions of the features by means of variations of rectangular-based shapes. Dasty ^4^ displays protein features from different sources as well as sequences and 3D structures, provindg an overview of the visualized protein. The Research Collaboratory for Structural Bioinformatics Protein Data Bank (RCSB-PDB) ^5^ mainly focuses on 3D structures for proteins but also includes feature visualization, showing the relationship between UniProt and PDB coordinates.

BioJS ^6^ is an open source JavaScript collection of components for visualization of biological data on the web. Here, we present *FeatureViewer* and its current extensions: *SimpleFeatureViewer* that simplifies the input data format, and *DasProteinFeatureViewer* that retrieves the input data from a web service. The *FeatureViewer* is a standard, portable BioJS component designed to easily render position-based annotations, a.k.a. features. The *FeatureViewer* component can be easily integrated into and controlled from other applications. As the *FeatureViewer* and its extensions have been developed within the BioJS framework, they result in a set of modular visual components displaying position-based annotations that can be integrated with other web applications in a standard manner. Modularity and easy integration differentiate these components from previous protein feature web based visualization efforts.

## The *FeatureViewer* component

The *FeatureViewer* component extensively uses the Raphaël javascript library ^7^ that renders Scalable Vector Graphics (SVG) objects in modern web browsers. The use of SVG allows the graphics to scale to any requested resolution and is portable across different computing platforms and viewing software.

**Figure 2.**
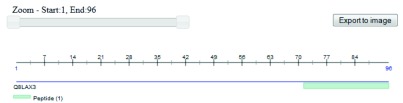
Visualization of a peptide using the *FeatureViewer* component.



The *FeatureViewer* component can be easily integrated into any web application by including its dependencies in the head section, e.g., jQuery ^8^ and Raphaël, and then instantiating the component within a JavaScript section. A special dependency for some images is required as they are used for the pop-up dialogue controls. The code below shows how to instantiate the component to create the visualization shown in Figure 2. A complete example and more information can be found at http://www.ebi.ac.uk/Tools/biojs/registry/Biojs.FeatureViewer.html. The *FeatureViewer* component has been tested with modern browsers such as Mozilla, Chrome, and Internet Explorer (IE); however, the image export option is not available in IE.

                    var json = {
  featuresArray:[{
   // configuration for each style
   nonOverlappingStyle:{heightOrRadius:10, y:56}
   ,centeredStyle:{heightOrRadius:40, y:75}
   ,rowsStyle:{heightOrRadius:10, y:157}
   // feature information
   ,featureLabel:’Elicitor peptide 3’
   ,evidenceText:’UniProt’
   ,typeCode:’SO:0001064’
   ,typeCategory:’Molecule processing’
   ,featureId:’UPKB_Q8LAX3_PEPTIDE_74_96’
   ,featureTypeLabel:’active_peptide’
   // Display information
   ,type:’rect’, fillOpacity:0.5, stroke:’#7DBAA4’
   ,height:10, r:10, y:56, x:529, cy:56, cx:529
   ,strokeWidth:1, fill:’#7DBAA4’, width:151
  }]
  ,segment:Q8LAx3
  ,legend:{/*omitted ...*/}
  ,configuration:{
   // centered style
   sizeYCentered:160, sequenceLineYCentered:95,
   ,verticalGridLineLengthCentered:172
   ,horizontalGridNumLinesCentered:6
   // similar non–overlaping & rows (omitted)
   ,style:’nonOverlapping’, nonOverlapping:true
   // ruler
   ,requestedStart:1, requestedStop:96
   ,rulerY:20, rulerLength:660, belowRuler:30
   ,pixelsDivision:50, aboveRuler:10
   // others
   ,sizeY:76, sizeX:700, rightMargin:20
   ,leftMargin:20, sequenceLineY:54
   ,sequenceLength:96, unitsize:6.875
   ,sizeYKey:210, verticalGridLineLength:66
   ,horizontalGridNumLines:2
   ,gridLineHeight:12
   ,verticalGrid:false, horizontalGrid:false
   ,verticalGridLineLengthRows:284
   ,horizontalGridNumLinesRows:8
   ,dasSources:’http://www.ebi.ac.uk/das
   –srv/uniprot/das/uniprot’
   ,dasReference:’http://www.ebi.ac.uk/das
   –srv/uniprot/das/uniprot’
   }
};
var myPainter = new Biojs.FeatureViewer ({
  target: ”YourOwnDivId”,
  json: json
});
                


### Options and data

**Figure 3.**
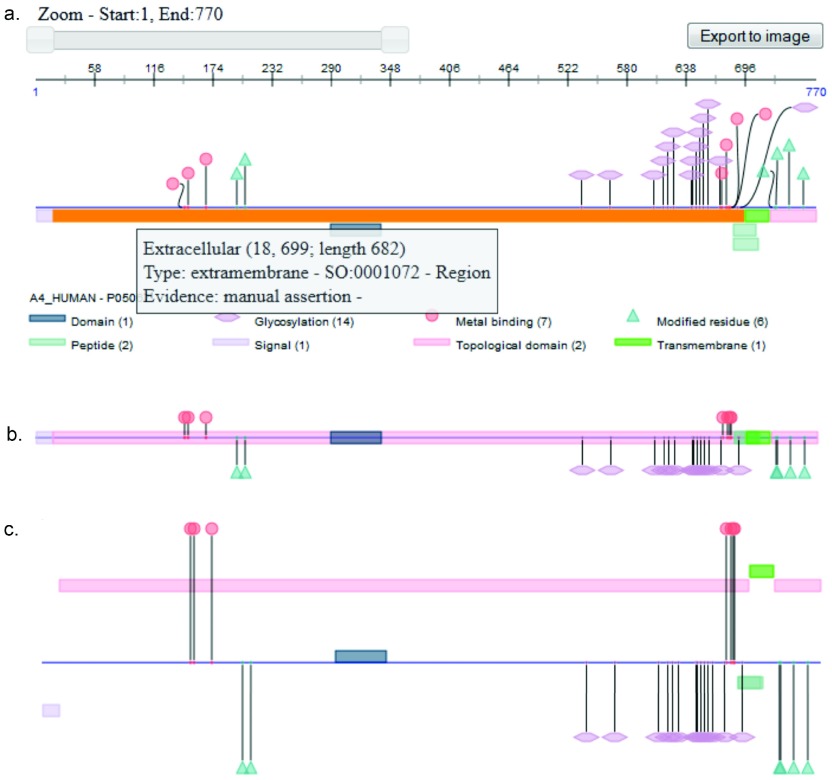
This visualization corresponds to the UniProt protein "Amyloid beta A4 protein" in the non-overlapping style; interactions such as shape dragging, tooltip, and selection as well as user controls such as zooming and image exporting are illustrated in this figure; **b**. shows the centralized view, while **c**. shows the by-rows view.

In order to instantiate the *FeatureViewer* component, some options should be defined. The mandatory options correspond to (i) a place holder named *target*, i.e., a DIV element in the web page where the annotations will be rendered, and (ii) a JSON object, named *json*, with the configuration, the protein identifier, the annotations, and the legend. *FeatureViewer* is a dummy component in the sense that it does not make any calculations about where to render the annotations, not even when the rendering style is changed; all the rendering information is provided in the *json* option. A comprehensive list of the elements in the *json* option is available at http://www.ebi.ac.uk/Tools/biojs/registry/Biojs.FeatureViewer.html. The *FeatureViewer* component includes three different layouts to display the features: all features centered, features grouped by type, and features organized in non-overlapping tracks, as shown in Figure 3.

### User controls

Additional options can also be specified in order to customize the user controls as well as interaction with the features. User controls include the zooming slider and the export-to-image button, as shown in the Figure 3a. The zooming slider allows users to hone in on a region of interest and view it in greater detail, making it possible to move from an overview aspect into a detailed one without navigating to a different page. The export-to-image button allows users to export the rendered features into an image that can embedded into a paper or presentation.

Different kinds of interaction are also possible. Events bound to rendered annotations include a mouse-over action that highlights and colors the "focused" feature. Click action is also supported. Clicking on a feature selects it so it will remain highlighted until another feature is selected; clicking an already selected feature will deselect it. Tooltips tied to each shape pop up and reveal additional information about the rendered annotations. Either shapes or lines can be used to display features covering one single amino acid; currently metal bindings can be rendered as circles, active sites as diamonds, lipidation as waves, glycosylation as hexagons, and other post translational modifications, i.e., modified residues, as triangles. When shapes are used, it is possible to drag them making it easier to distinguish one from another when they are clustered.

### Extensions

**Figure 4.**
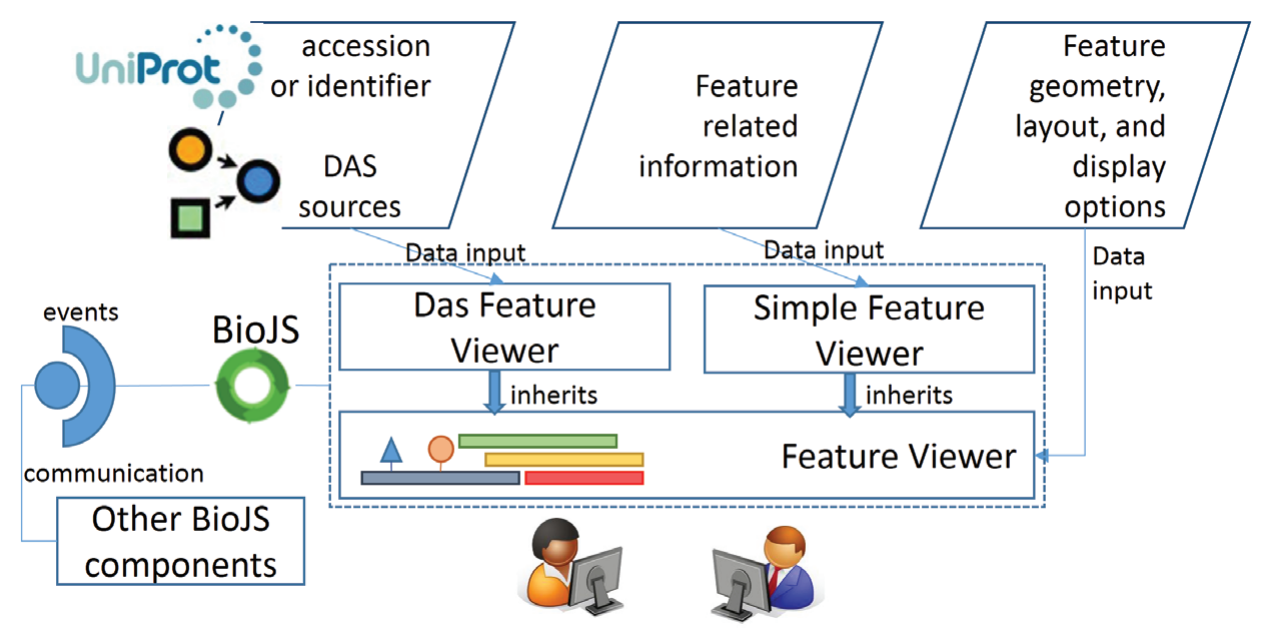
This graphic shows the *FeatureViewer* component and its extensions as well as the variations on the required input data. It also illustrates how BioJS handles the interaction with other components by means of events.

In order to make it easier for both developers and users to work wiht the *FeatureViewer*, two extensions are also provided. *SimpleFeatureViewer* simplifies the required features data while *DasProteinFeatureViewer* uses a web service to retrieve the features data. Figure 4 shows the three components in the Feature Viewer family.

As *FeatureViewer* requires highly detailed information in order to display the features, a simpler version, the *SimpleFeatureViewer* component, builds on top of it. This simplified version takes care of calculating the configuration options as well as the localization of the features; thus, developers using this version can focus on the actual data rather than on intricate details regarding styles, pixels, and coordinates. However, only the non-overlapping tracks style is supported by this component. The main advantage of this component is that its data structure is much simpler than the one required for *FeatureViewer*, as observed in the following code excerpt.



                        var myFT = [
  {
   featureId:’UNIPROTKB_Q8LAX3_PEPTIDE_54_96’,
   featureStart:54, featureEnd:96,
   typeLabel:’Peptide’, typeCode:’SO:0001064’, 
   featureLabel:’Elicitor peptide 3’,
   typeCategory:’Molecule processing’,
   evidenceText:’UniProt’, evidenceCode:’ ’,
   color:’blue’
  },
  {
   featureId:’UNIPROTKB_Q8LAX3_PEPTIDE_74_96’,
   featureStart:74, featureEnd:96,
   typeLabel:’Active Site’, 
   typeCode:’SO:0001064’
   featureLabel:’Elicitor peptide 3’,
   typeCategory:’Molecule processing’,
   evidenceText:’UniProt’, evidenceCode:’ ’,
   type:’diamond’
  },
  {
   featureId:’UPKB_Q8LAX3_DISULFID_75_96’,
   featureStart:75, featureEnd:96,
   typeLabel:’Active Site’,
   typeCode:’SO:0001064’
   featureLabel:’Elicitor peptide 3’,
   typeCategory:’Molecule processing’,
   evidenceText:’UniProt’, evidenceCode:’ ’,
   color:’#33FF66’,
   type:’bridge’
   }];
var myPainter =
   new Biojs.SimpleFeatureViewer ({
   target:’YourOwnDivId’,
   sequenceId:’a4_human’,
   sequenceLength:770,
   features:myFT
});
                    


This component requires a place holder, a sequence identifier, a sequence length, and a features array; the width in pixels to be used to rendered the protein features can also be defined by using the option *imageWidth*. The features array contains information for each feature to be displayed including, for instance, identifier, start and end positions in the sequence, label, and color among others. More information is available at http://www.ebi.ac.uk/Tools/biojs/registry/Biojs.SimpleFeatureViewer.html.

 A second extension, the *DasProteinFeatureViewer*, makes use of a web service that retrieves data from Distributed Annotation System (DAS) sources. DAS defines a communication protocol used to exchange annotations on gene or protein sequences ^9^. Multiple protein databases provide their data following the DAS principles, for instance UniProt and InterPro ^10^. For this extension, no information about the features themselves is required as such details will be retrieved from the web service, as shown in the code below.



                        var myPainter =
  new Biojs.DasProteinFeatureViewer({
   target: ”YourOwnDivId”,
   segment: ”a4_human”
});
                    


Additional options allow developers to specify the protein identifier, the DAS sources, the feature types – e.g., domain, chain, variant, etc., the rendering style, the image width, and some others. In order to avoid cross-domain problems, a proxy can also be specified. The feature types used by this component are those defined by UniProt, which is also used as the reference DAS source, i.e., the one providing the protein sequence. More information available at http://www.ebi.ac.uk/Tools/biojs/registry/Biojs.DasProteinFeatureViewer.html

## Use case

**Figure 5.**
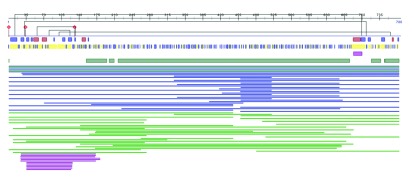
*FeatureViewer* is used by the PredictProtein service to show a stack of predicted structure and function features.

The PredictProtein service ^3^ integrates multiple algorithms that either retrieve from curated databases or automatically predict aspects of protein structure and function. Many of the predictions provided by the methods are mapped to positions within the protein. In order to easily highlight patterns, compare predictions, and cross-validate results, the PredictProtein interface lays out the predicted annotations in data tracks, i.e., in separate rows, each row presenting different predicted features. Data tracks are laid one under the other and enable the quick overview of some of the prominent features of the protein e.g., a cluster of binding sites close to the N-terminal or the count of trans-membrane regions. Figure 5 shows the implementation of the *FeatureViewer* component used for the PredictProtein service.

## Conclusions

The *FeatureViewer* component and its extensions, *SimpleFeatureViewer* and *DasProteinFeatureViewer*, provide a platform to visualize position-based biological data easily and efficiently. *FeatureViewer*, like any other BioJS component, can be easily integrated with other web components or extended to have greater functionality than the one shown here. We expect this component to be particularly useful to developers and users alike, requiring little technical knowledge for its full functioning.

## Software availability

Zenodo: BioJS Feature Viewer, doi: 10.5281/zenodo.7719 ^11^

GitHub: BioJS, https://github.com/biojs/biojs/
