# Supplementary material for: FeatureViewer, a BioJS component for visualization of position-based annotations in protein sequences
Source: F1000Res. 2014 Apr 9;3:47. Originally published 2014 Feb 13. [Version 2] doi: 10.12688/f1000research.3-47.v2 (PMC3983936; doi:10.12688/f1000research.3-47.v2)
Supplement: Peer review history

# Peer review: Referee response for version 1

**Published:** 2014-03-18

**Authors:** Andreas Prlic

**Affiliations:** 1 San Diego Supercomputer Center, University of California, San Diego, CA, USA

There are currently only a few approaches available for visualising protein features on the web. The *BioJS FeatureViewer* is a welcome addition to the set of tools available to web developers. The fact that it is available as a BioJS component should make it interesting as a re-usable web site element. However, I find that some of the already existing approaches provide a representation of features that I (subjectively) find graphically more refined and that go beyond simple boxes, circles, and triangles to represent sequence annotations.

Minor modifications:

I feel that the current version of this manuscript does not adequately summarise the already available approaches for visualising protein features. To list a few already available protein feature viewers:

- **Pfam** (is already being mentioned in the manuscript), shows the location of Pfam domains on protein sequences. It has currently one of the graphically most appealing views and can show more than one sequence per page (e.g. http://pfam.sanger.ac.uk/family/Piwi#tabview=tab1)

- **PDBsum** provides a graphical summary of secondary structure and pfam domains for protein sequences (e.g. https://www.ebi.ac.uk/pdbsum/1cdg and in large: https://www.ebi.ac.uk/thornton-srv/databases/cgi-bin/pdbsum/GetPage.pl?pdbcode=1cdg&template=wirpfam.html&r=wiring&l=1&chain=A&pfam=TRUE&form=TRUE&nres=150)

- **DASty** has been developed for several years at the EBI (in fact there seems to be some shared co-authorship) and provides a feature view that is based on DAS and can also map annotations to 3D (e.g. https://www.ebi.ac.uk/dasty/client/index.html?q=P05067)

- **RCSB Protein Feature View**: shows an SVG image for a full-length protein sequence from UniProt and how it corresponds to PDB entries. It loads annotations from external databases (e.g. http://www.rcsb.org/pdb/protein/P43379) and is available as open source from https://github.com/rcsb/proteinfeatureview

- **RCSB Sequence Page**: can show the relationship between UniProt and PDB coordinates on a per-residue level. Also can map annotations to 3D ( e.g. http://www.rcsb.org/pdb/explore/remediatedSequence.do?structureId=1CDG).

I have read this submission. I believe that I have an appropriate level of expertise to confirm that it is of an acceptable scientific standard.

# Response

**Published:** 2014-04-03

**Authors:** L. Garcia

**Affiliations:** EMBL-EBI, UK

Dear Andreas,

Thanks for your review, it has been useful to improve our work. Please see below our responses.

**Response: **We have included a new paragraph in the Introduction in order to cover other efforts on web visualizations for protein sequence annotations. We have included those related to protein sequence features in general rather than specialized on secondary or 3D structures.

# Peer review: Referee response for version 1

**Published:** 2014-02-25

**Authors:** Jim Procter

**Affiliations:** 1 Computational Biology, College of Life Sciences, University of Dundee, Dundee, UK

***Summary***

This *F1000Research* article describes an ensemble of JavaScript components designed for bioinformatics web developers that allow the retrieval, layout and display of positional annotation on a 1D coordinate system, such as a protein or nucleotide sequence. Special support is provided for the display of protein positional annotation retrieved from the Distributed Annotation System (requires some server-side configuration), and the system provides standard glyphs and shading styles to allow active sites and common types of post-translational modifications to be effectively displayed. Importantly, the components employ the BioJS framework, which allows them to exchange messages with other BioJS biological data visualization components (as well as any jquery based module) to facilitate the creation of rich, interactive web interfaces.

***Using the component***

The feature viewer component example page demonstrates that it provides a clean static visualization of the positional annotation as it appears on a coordinate system (complete with annotation legend). Online documentation supports the authors’ statement that the plugin is highly configurable: attributes are provided to control practically every aspect of an annotation's appearance, and the surrounding coordinate space and additional decorators'. A few minutes spent interacting with the demonstration, however, shows that there is room for improvement:

**(1) Suggested improvement**. Once the zoom control is adjusted to focus in on a smaller region, it doesn't allow the user to sweep across the coordinate space (i.e. by click-dragging the visible range). From a developer perspective, I would also expect the component to raise BioJS events to inform other components about the change in 'Region Of Interest' to allow them to respond in kind.

**(2) Suggested improvement.** The authors mention that feature glyphs can be dragged to facilitate inspection in crowded regions. It is unclear how useful this capability is – since once dragged, any change in view results in the feature glyph’s shape being reset, and in crowded regions, the user must – by definition – move many features to examine the precise location of annotation. User modified glyph layout should – at the very least – be preserved on changes of scale. There may also be wisdom in including a force-directed layout algorithm to better optimize placement of nearby glyphs in response to manual adjustment of any particular one.

 

***Developing with the component(s)***

Apart from choosing appropriate values for the vast array of attributes and annotation display settings that *FeatureViewer* supports (more on that later), the most onerous aspect of deploying *FeatureViewer* is keeping track of the array of dependencies it requires. However, JavaScript module dependency management is a moving target, and I make the recommendation below with that in mind:

 

**(3) Suggested improvement**. I **strongly recommend **that the authors provide examples that employ a client-side JavaScript package management system such as 'RequireJS' (see http://jquerysbestfriends.com for some other tips about more sanitary methods of deploying jquery). Dependency management is important, and demonstrating good practice will allow these tools to be more widely adopted, and more effectively maintained.

One aspect of this paper that was not immediately clear on first reading was the functional relationship between *FeatureViewer* – which renders annotation layouts into SVG, and the helper components SimpleFeatureRenderer and DASFeatureRenderer.

**(4) Suggested revision/reviewer contribution**

A diagram such as the one I uploaded to FigShare here:

http://figshare.com/articles/The_BioJS_FeatureViewer_components_for_sequence_annotation_retrieval_layout_and_display/942422

may help readers of this article more effectively grasp the different functionalities provided by the components in this system.

 

***Specific revisions/corrections***

 

**(5) Required revision**

In the abstract, the authors claim: "To our knowledge, this is the first client-side modular component to visualize position-based annotations that can be integrated into other web applications in a standard manner."

This is not quite correct. There have been other client-side modular web components that have been created for the visualisation of positional annotation, although some of the co-authors may have, for modesty reasons omitted mentioning the fact. In this case, Dasty3 and its forerunner, Dasty2 both provided a self-contained (ie modular) component that could be controlled through javascript and embedded in a more complex web page.

 

Here, I recommend the authors emphasise more strongly the key novelty in this work. For example: " **because these components are built with the BioJS framework**, they are the first modular visualization components for the display of position-based annotation that can be integrated with other web applications in a standard manner."  

**(6) Please revise the abstract for clarity**

"This component is highly flexible and customizable, allowing the presentation of annotations by rows, all centered, or distributed in non-overlapping tracks. It uses either lines or shapes for sites and rectangles for regions."

Point 1. I'm not sure what presenting annotations "all centered" actually means!

Point 2. (optional). The authors might consider providing a biological example. This would help the lay-reader understand what the tool does without already having deep knowledge of protein sequence annotation visualization.

**(7) Please revise the following sentence for clarity**

 

The authors state that DasFeatureViewer can employ different glyphs for particular types of protein sequence annotation, including post translational modifications (PTMs).

 

Point 1. Suggested revision in ***bold italics***

“Either shapes or lines can be used to display features covering one single amino acid; currently metal bindings can be rendered as circles, active sites as diamonds, lipidation as waves, glycosylation as hexagons, and [ ***other***] post translational modifications as triangles.“

The reason for this revision is that Lipidation and glycosylation are, of course, both PTMs. The authors could also mention here that triangles are used as the default for other types of ‘MOD_RES’ type PTM (glycation, hydroxylation, phosphorylation, etc).

 

**(8) Minor typos/grammar**

 

Point 1. In the Introduction section "Particularly in the case of proteins, the Pfam database identifies, marks-up, and characterizes different functional regions within a given protein." THe word 'Particularly' is unnecessary.

Point 2. In the following sentence (in the Introduction section): "The largest pool of reviewed and automatically annotated proteins provided by the UniProt Consortium also contains position-based annotations for structural regions, modified residues, and functional sites among others." The authors probably mean to make the following statement (revision in ***bold italics***):

"The largest pool of reviewed and automatically annotated proteins ***is ***provided by the UniProt Consortium ***. It*** also contains position-based annotations .."

Point 3. Again, revising for clarity (in the Introduction section) (revision in ***bold italics***):

"Finally, ***protein feature prediction*** methods such as those integrated into PredictProtein provide ***position-based*** feature.."

Point 4. In the next paragraph: "and *DasProteinFeatureViewer* that retrieves the input data from a web service." There is a missing space between 'and' and 'DasProteinFeatureViewer'

 

Point 5. In the second paragraph of “The *FeatureViewer* component" section “The code below shows how to instantiate the component; the corresponding visualization is shown in Figure 1.” This sentence is perhaps more cleanly stated as:

“The code below shows how to instantiate the component to create the visualization shown in Figure 1”.

 

**(9) Typos in Web Resources**

Please fix the missing ‘ ***e***’ on this page: http://www.ebi.ac.uk/Tools/biojs/registry/Biojs.DasProteinFeatureViewer.html

"This component uses a DASProtein web service that builds the JSON data object used by F ***e***atureViewer component. Version 1.0.0."

I have read this submission. I believe that I have an appropriate level of expertise to confirm that it is of an acceptable scientific standard.

# Response

**Published:** 2014-04-03

**Authors:** L. Garcia

**Affiliations:** EMBL-EBI, UK

Dear James,

Thanks for your review, it has have been useful to improve our work. We have tried to addressed all you comments, however those related to the component itself, i.e., JavaScript code, will be taken into account for a new version of the software, and those related to BioJS in general will be sent to BioJS core developers. Please see our responses below.

**Response to (1) and (2): **We are currently working on an improved component to visualize protein sequence annotations. We have made notes about this suggestions and will take them into account for the new visualization. Unfortunately, such improvements are not yet ready to be integrated into the public BioJS GitHub repository.

**Response to (3):** We agree with the reviewer on the convenience of using dependency management for BioJS components. As we think it will impact not just this component but any other currently in BioJS, we have initiated a discussion on how to improve this aspect in BioJS, but no decision has been made yet. We will take it into account for the new visualization we are working on.

**Response to (4):** We have included a figure similar to the proposed one by the reviewer at the beginning of the 'Extensions' section. It introduces the extensions and indeed, as mentioned by the reviewer, helps the reader to understand the relation across the three components.

**Response to (5): **We have included a new paragraph in the Introduction in order to cover other efforts on web visualization for protein sequence annotations, covering not only Dasty but others as well. We have also emphasized the novelty of this work as suggested by the reviewer.

**Response to (6): **We have simplified the abstract.

**Response to (7 and 8): **We have followed the reviewer’s suggestions.

**Response to (9): **We have added the missing ‘e’ and it will be included in the next BioJS registry update.

Regards
